# Post Meal Energy Boluses Do Not Increase the Duration of Muscle Protein Synthesis Stimulation in Two Anabolic Resistant Situations

**DOI:** 10.3390/nu11040727

**Published:** 2019-03-29

**Authors:** Laurent Mosoni, Marianne Jarzaguet, Jérémie David, Sergio Polakof, Isabelle Savary-Auzeloux, Didier Rémond, Dominique Dardevet

**Affiliations:** Unité de Nutrition Humaine, INRA, Université Clermont Auvergne, UMR1019, F-63000 Clermont-Ferrand, France; laurent.mosoni@inra.fr (L.M.); marianne.jarzaguet@inra.fr (M.J.); jeremie.david@inra.fr (J.D.); sergio.polakof@inra.fr (S.P.); isabelle.savary-auzeloux@inra.fr (I.S.-A.); didier.remond@inra.fr (D.R.)

**Keywords:** aging, catabolic state, anabolic resistance, protein synthesis, energy bolus

## Abstract

Background: When given in the long term, whey proteins alone do not appear to be an optimal nutritional strategy to prevent or slow down muscle wasting during aging or catabolic states. It has been hypothesized that the digestion of whey may be too rapid during a catabolic situation to sustain the anabolic postprandial amino acid requirement necessary to elicit an optimal anabolic response. Interestingly, it has been shown recently that the duration of the postprandial stimulation of muscle protein synthesis in healthy conditions can be prolonged by the supplementary ingestion of a desynchronized carbohydrate load after food intake. We verified this hypothesis in the present study in two different cases of muscle wasting associated with anabolic resistance, i.e., glucocorticoid treatment and aging. Methods: Multi-catheterized minipigs were treated or not with glucocorticoids for 8 days. Muscle protein synthesis was measured sequentially over time after the infusion of a ^13^C phenylalanine tracer using the arterio-venous method before and after whey protein meal ingestion. The energy bolus was given 150 min after the meal. For the aging study, aged rats were fed the whey meal and muscle protein synthesis was measured sequentially over time with the flooding dose method using ^13^C Valine. The energy bolus was given 210 min after the meal. Results: Glucocorticoid treatment resulted in a decrease in the duration of the stimulation of muscle protein synthesis. The energy bolus given after food intake was unable to prolong this stimulation despite a simultaneous increase of insulin and glucose following its absorption. In old rats, a similar observation was made with no effect of the energy bolus on the duration of the muscle anabolic response following whey protein meal intake. Conclusions. Despite very promising observations in healthy situations, the strategy aimed at increasing muscle protein synthesis stimulation by giving an energy bolus during the postprandial period remained inefficient in our two anabolic resistance models.

## 1. Introduction

Skeletal muscle size and function are directly related to the amount of muscle protein content and quality. Muscle protein homeostasis is ensured by continuous turn-over, including the degradation of non-functional muscle proteins which are replaced by the synthesis of new ones. This equilibrium between muscle protein synthesis and breakdown varies throughout the day. During the post-absorptive period, in the absence of food intake, muscle proteolysis exceeds synthesis to provide essential amino acids to other organs, whereas an anabolic response is initiated when muscle protein synthesis is higher than proteolysis after food intake [1]. It is now well-established that the postprandial anabolic response in skeletal muscle occurs through the stimulation of protein synthesis initiated by increased essential amino acid bioavailability, whereas the decrease in proteolysis mainly occurs through the stimulation of insulin secretion in response to feeding [2,3]. Although decreased physical activity has been identified as a cause, muscle atrophy has also been explained by the presence of anabolic resistance following food intake in several physio-pathological catabolic states as diverse as aging, cancer, and disuse [4,5,6,7,8,9,10]. In each case, increasing dietary protein intake above the current recommended dietary allowances (RDA at 0.8 g/kg/day) [11] has been proposed to overcome the anabolic resistance observed. Among dietary proteins, rapidly digested and leucine-rich proteins (i.e., whey) have been shown to be the most efficient for the acute stimulation of muscle protein synthesis during aging, disuse or glucocorticoid treatment when compared to casein, a protein with lower leucine content and whose digestion rate is slower [9,12,13,14]. However, when given in the long-term, whey proteins alone do not appear to be an optimal nutritional strategy to prevent or slow down muscle wasting during aging [15,16] or catabolic states [10,17,18,19]. This could be explained by the nature and intensity of the catabolic state but also by the fact that the digestion of whey may be too rapid during a catabolic situation to sustain the anabolic postprandial amino acid requirement necessary to elicit an optimal anabolic response [20]. Indeed, the stimulation of muscle protein synthesis has a defined and limited duration during the postprandial period [21,22]. Atherton et al. showed that whey ingestion is only able to stimulate muscle protein synthesis for 2 h [21]. The leucine content of a complete meal drives peak activation but not the duration of skeletal muscle protein synthesis [23]. Interestingly, it has been shown recently that the duration of the postprandial stimulation of muscle protein synthesis in healthy conditions could nevertheless be prolonged by maintaining the cellular energy status with the supplementary ingestion of a desynchronized carbohydrate load after food intake (±150 min) [24]. Such a prolongation would be very useful to slow down the loss of muscle mass during aging, or in other catabolic situations. To our knowledge, the use of such an energy bolus at a precise time after a meal has never been tested in catabolic situations. Thus, the present study aimed to determine whether an energy bolus given 30 min after peak postprandial muscle protein synthesis stimulation could also increase muscle protein synthesis during a catabolic situation. We tested two catabolic situations: glucocorticoid treatment in young mini pigs, and chronic loss of muscle mass during aging in the rat. Unfortunately, in both situations, the energy bolus was unable to re-stimulate muscle protein synthesis.

## 2. Materials and Methods

In the present manuscript, we studied two different muscle wasting conditions for which we have clearly described an anabolic resistance in skeletal muscle following food intake. In these two studies, we showed that whey, a rapidly digested protein rich in leucine, was more efficient than casein at the same protein content for (re)initiating an anabolic response, mainly by increasing muscle protein synthesis postprandially [9,10]. Thus, some of these results which represent the “control” groups in these manuscripts have been already published. In the present publication, the original results are the results in the same conditions, but associated with the desynchronized energy bolus. 

### 2.1. Glucocorticoid Treatment Study

#### 2.1.1. Animal Housing, Surgery and Ethics Statement

The present study was approved by the Animal Care and Use Committee of Auvergne (CEMEA Auvergne; Permit Number: CE 68-12) and the Ministère de l’Enseignement Supérieur et de la Recherche (no. 02125.02) and described in full by Revel et al. [10]. For the study, 18 adult male Yucatan mini pigs (averaging 20 kg) were housed individually in subject pens (1 × 1.5 m) in a ventilated room with controlled temperature (21 °C). They were fed twice daily with 220 g/d of a concentrated feed containing 16% protein, 1% fat, 4% cellulose, and 5% ash (Porcyprima; Sanders Centre Auvergne, Aigueperse, France) and had free access to water. Three weeks before the experiment, the minipigs went into surgery and were fitted with catheters in the inferior cava vein and the aorta. For the cava vein, the catheter was inserted just downstream of the junction with the iliac veins. A transit time ultrasonic blood flow probe (6 mm probe, R-series; Transonic Systems, Inc., Ithaca, NY, USA) was implanted around the distal aorta 1–2 cm before it splits into the iliac arteries. Catheters and probe cables were exteriorized through the skin of the right flank of the animal. A minimum of 2 weeks was allowed for recovery from surgery before initiating the experiment. Surgical procedures, as well as post-surgical care, were described previously in detail by Rémond et al. [25].

#### 2.1.2. Tracer Infusion Procedures

On the day of the experiment, after an overnight fasting period, the animals were separated into 3 groups (*n* = 6 per group): control group (WHEY CONTROL), glucocorticoid treated group (WHEY DEXA), and the glucocorticoid group with a desynchronized energy bolus during the postprandial period (WHEY DEXA BOLUS). For all groups, after basal blood samples were withdrawn from the artery and iliac vein, a priming dose of labeled [ring U-^13^C] L-Phenylalanine (4.2 µmol∙kg^−1^) was injected and then [ring U-^13^C] L-Phenylalanine was infused (4.2 µmol.kg^−1^∙h^−1^) for 550 min through the hepatic vein. During the first 150 min, the animals remained deprived of food and samples of arterial and iliac venous blood were simultaneously withdrawn at 90, 120, and 150 min, and represented the post-absorptive period (PA). At t = 150 min, the animals consumed a test meal (250 g) containing whey as a protein source (13%), as well as lipids (6%), and carbohydrates (57%). The meal was entirely consumed within 10–15 min, arterial and iliac venous blood was sampled every 30 min during the remaining 400 min (postprandial period, PP). The ultrasonic blood flow probe was used to record blood flow continuously throughout the whole experiment. For the glucocorticoid-treated group (WHEY DEXA), 8 days before the experiment, the animals received a dose of 0.4 mg/kg/day of dexamethasone added to the feed every morning (0.15 mg/kg) and evening (0.25 mg/kg). Food intake was not modified by the treatment. The animals were subjected to the same tracer infusion protocol described above except that dexamethasone was consumed with the test meal. The WHEY DEXA BOLUS group followed the same protocol except that 150 min after the test meal ingestion (30 min after the expected peak of protein synthesis observed in our previous experiment [10]), the animals were allowed to consume 100 g of glucose/ saccharose (50/50) to initiate an energy bolus similar to 70% of the carbohydrate content of the test meal, as described by Wilson et al. [24]. In the experiment performed by Wilson et al., the bolus was similar to 100% of the carbohydrate content of the test meal and was given 45 min after peak muscle protein synthesis.

#### 2.1.3. Samples Analysis

The plasma ^13^C enrichment of phenylalanine was measured by gas chromatography–mass spectrometry (GC–MS, model HP5975C/7890A, Agilent, Santa Clara, CA, USA) with the use of tertiary-buthyldimethylsilyl derivatives and by ion monitoring with m/Z 336 and 342, as previously described [10,26].

Plasma concentrations of amino acids were determined by ion exchange chromatography on deproteinized samples [10]. Plasma glucose was assayed using an enzymatic method on an autoanalyzer (Pentra 400, Horiba, Montpellier, France) and the insulin concentration was assayed by ELISA (Mercodia, Uppsala, Sweden).

#### 2.1.4. Calculations

Muscle protein synthesis was calculated using the arterio-venous differences method according to the equations previously described by Bruins et al. [27] and Paddon-jones et al. [28]. Phenylalanine was selected to represent amino acid kinetics because it is neither produced nor metabolized in skeletal muscle [29]. In this tissue, the disposal and production of phenylalanine reflect protein synthesis and protein breakdown. The complete and detailed equations used to calculate muscle protein synthesis were described by Revel et al. [10]. 

#### 2.1.5. Statistics and Analysis of Results

Data are presented as means ± SE. Repeated time variance analyses were performed to test the effect of time, status (WHEY CONTROL, WHEY DEXA, WHEY DEXA BOLUS) and interaction time × status. LSD post-hoc tests were used to compare mean values at each time (Statview, SAS Institute, Cary, NC, USA).

### 2.2. Aging Study

#### 2.2.1. Animals and Diets

The present study was approved by the Animal Care and Use Committee of Auvergne (CEMEA Auvergne; Permit Number: C2EA-02) and the Ministry of Higher Education and Research (no. 2016101911586999). Twenty-month-old male Wistar rats (Charles River, L’Arbresle, France) were housed individually and kept in a controlled environment (temperature maintained at 22 °C; 12:12 light: dark cycle). The average weight of the rats was close to 600 g. After an adaptation period during which the animals were fed regular chow (Safe A04, Augy, France), they were divided into 2 groups, fasted overnight and fed the next morning with a meal (6 g). The majority of this amount (>60%) was consumed within the first 30 min and withdrawn after 1 h if any food remained uneaten. One group received the meal made with whey (13%) as a protein source (+ 6% lipids and 71% carbohydrates) and the second group received the same meal but also 4.5 g of glucose/sucrose (50/50), which was similar to 104% of the amount of carbohydrate in the test meal 210 min after the beginning of the meal. Once again, our aim was to mimic the study of Wilson et al. [24] but in heavier and older rats. Our bolus was given 30 min after peak muscle protein synthesis. The animals were then sacrificed before (0) and 90, 125, 180, 240 and 270 min (only for the energy bolus group) after the beginning of the meal under 4% isoflurane anesthesia (*n* = 10/time point). An abdominal incision was made and blood was withdrawn from the abdominal aorta with syringes containing EDTA. The gastrocnemius muscles were rapidly removed, weighed and freeze-clamped in liquid nitrogen, and stored at −80 °C. Regarding the muscle protein synthesis measurement, 40 min before the sacrifice, animals were injected intravenously with L-valine (150 μmol per 100 g body weight) containing 100% L-[1-^13^C] valine (Euriso-Top) (see [9] for the detailed protocol).

#### 2.2.2. Analytical Procedures

Glucose concentrations were measured enzymatically using commercial kits (Horiba, Montpellier, France). Plasma insulin levels were also assessed using a commercial ELISA kit (Mercodia, Uppsala, Sweden). To measure protein synthesis, the muscles were powdered in liquid nitrogen in a ball mill (Dangoumeau, Prolabo, Paris, France). A 200 mg aliquot of frozen muscle powder was homogenized in 2 mL of 10% trichloroacetic acid (TCA). Proteins were hydrolyzed in 6 N HCl at 110 °C for 48 h. HCl was removed by evaporation and amino acids purified by cation exchange chromatography. The enrichment of [1-^13^C] valine in muscle proteins was measured on the basis of its N-acetyl-propyl derivatives by gas chromatography–combustion-isotope ratio mass spectrometry (GC–C-IRMS). The plasma ^13^C enrichment of valine was measured by gas chromatography–mass spectrometry (GC–MS, model HP5975C/7890A, Agilent, Santa Clara, CA, USA) with the use of tertiary-buthyldimethylsilyl derivatives (see [9] for the detailed protocol). The absolute synthesis rate (ASR: total muscle protein synthesized) was calculated from the product of the protein fractional synthesis rate (FSR) and the protein content of the tissue, and expressed in mg/d. FSR (in %/day) was calculated from the formula: FSR = Sb × 100/Sa × t, where Sb is muscle protein-bound [1-^13^C] valine enrichment (minus natural basal enrichment of protein), Sa is the mean enrichment of plasma valine during tracer incorporation, and t is the incorporation time in days calculated between the time of tracer injection and the time of muscle sampling, i.e., “PA” between −40 min and 0 min; “90 min” between 50 and 90 min; “125 min” between 85 and 125 min; “180 min” between 140 and 180 min, “240 min” between 200 and 240 min and “270 min” between 230 and 270 min. The mean Sa enrichment was the Sa (t1/2) value calculated from the linear regression obtained in the tissue between the time of injection and time t.

#### 2.2.3. Statistics and Analysis of Results

Data are presented as means ± SE. Separate groups of animals were used each time; thus a repeated time variance analysis could not be performed. We used a two-way variance analysis to discriminate between the effect of time of measurement, and the effect of nutritional status (WHEY or WHEY BOLUS). The interaction was not testable since, by construction, the bolus was given at time 210 min and affected only time 240 and time 270. LSD post-hoc tests were performed to compare mean values at each time (Statview, SAS Institute, Cary, NC, USA).

## 3. Results

### 3.1. Minipigs Treated with Glucocorticoids

#### 3.1.1. Plasma Glucose, Insulin, and Arterial Amino Acid Concentrations

**Figure 1:**
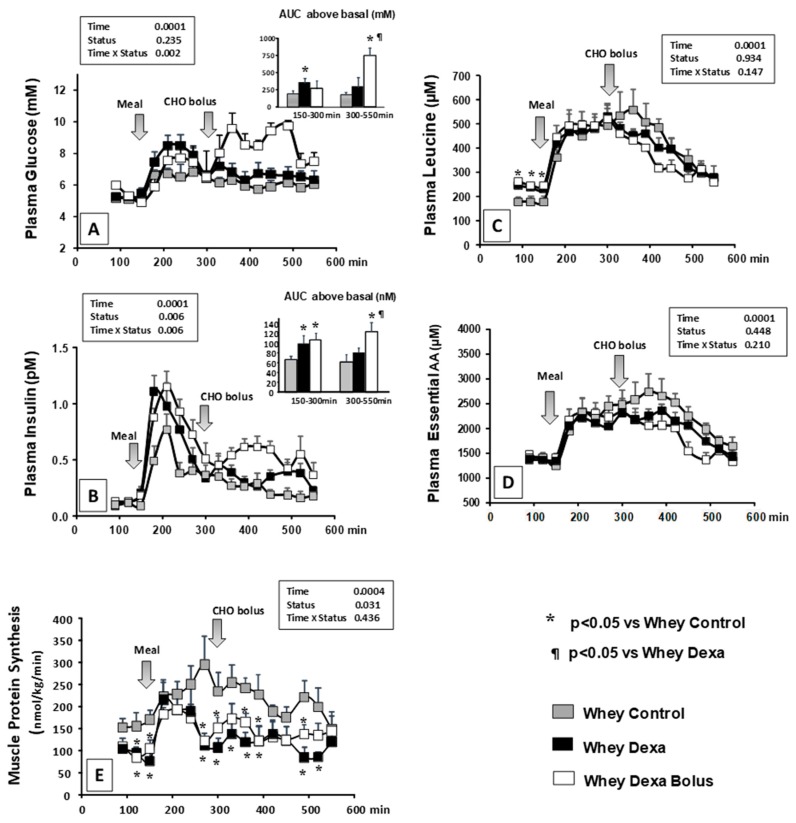
Plasma glucose (**A**), insulin (**B**), leucine (**C**) and essential amino acids (**D**) in control (WHEY CONTROL), in glucocorticoid-treated (WHEY DEXA) and in glucocorticoid-treated with the carbohydrates (CHO) bolus (WHEY DEXA BOLUS) minipigs. The areas under the curve (AUC) for glucose and insulin are presented in inserts. Postprandial AUCs were calculated by subtracting the post-absorptive values (t = 90–150 min) to each postprandial values. (**E**): Muscle protein synthesis kinetics for the post-absorptive period (before t = 150 min) and after the meal (after 150 min) in control (WHEY CONTROL), in glucocorticoid-treated (WHEY DEXA) and glucocorticoid-treated + carbohydrate (CHO) bolus (WHEY DEXA BOLUS) minipigs. The CHO bolus was given at t = 300 min. Data are presented as means ± SEM. (*n* = 6).

After the whey-based meal, an increase in both glucose and insulin was observed (Figure 1A,B). Plasma glucose increased rapidly (1.2-fold between 180–240 min) and gradually decreased to the basal values throughout the remaining postprandial period (300–550 min). Insulin was secreted rapidly with maximum stimulation at 210 min (7–8-fold), and then insulin decreased back to the basal values throughout the rest of the postprandial period. 

Plasma insulin and glucose increased in WHEY DEXA minipigs during the early postprandial period (150–300 min), when compared to the control situation. During the late postprandial period (300–550 min), no significant differences were observed for either plasma insulin or glucose between the WHEY CONTROL and the WHEY DEXA minipigs (Figure 1A,B). When the glucocorticoid-treated mini pigs received the energy bolus at 300 min (WHEY DEXA BOLUS), a significant increase in both plasma glucose and insulin was recorded in the late postprandial period (300–550 min) when compared to the two other groups: WHEY CONTROL and WHEY DEXA (Figure 1A,B). 

Plasma leucine also increased rapidly (approximately 2-fold) after the WHEY CONTROL meal intake (Figure 1C). This difference was maintained during the first two-thirds of the postprandial period, then finally decreased rapidly during the late postprandial period (>400 min) (Figure 1C). The same pattern was recorded for the essential amino acids (EAA) (Figure 1D). 

Post-absorptive leucine concentrations were significantly increased after the glucocorticoid treatment (time 0, 90 and 120 min: +35–40%) (Figure 1C). By contrast, the glucocorticoid treatment did not significantly alter plasma leucine and EAA kinetics following whey meal intake (Figure 1D). When the energy bolus was given at 300 min, no significant changes were recorded in leucine or EAA kinetics in the late postprandial period (300–550 min) in the WHEY DEXA BOLUS when compared to the WHEY CONTROL and WHEY DEXA groups (Figure 1D).

#### 3.1.2. Muscle Protein Synthesis

When fed the whey meal, muscle protein synthesis increased to a peak at 240 min after which it decreased slowly until the end of the postprandial period studied (Figure 1E). In the WHEY DEXA group, muscle protein synthesis was significantly lower in the post-absorptive state and was only transiently significantly stimulated by the whey protein during the early part of the post-prandial period (150–270 min) (Figure 1E). In the late post-prandial period, muscle protein synthesis remained significantly lower in the WHEY DEXA group when compared to the WHEY CONTROL group. When the energy bolus was given at 300 min, no modification of muscle protein synthesis was recorded when compared to the WHEY DEXA group and it stayed significantly different to the values recorded in the WHEY CONTROL group (Figure 1E).

### 3.2. Aging Study

#### Plasma Glucose and Insulin

**Figure 2:**
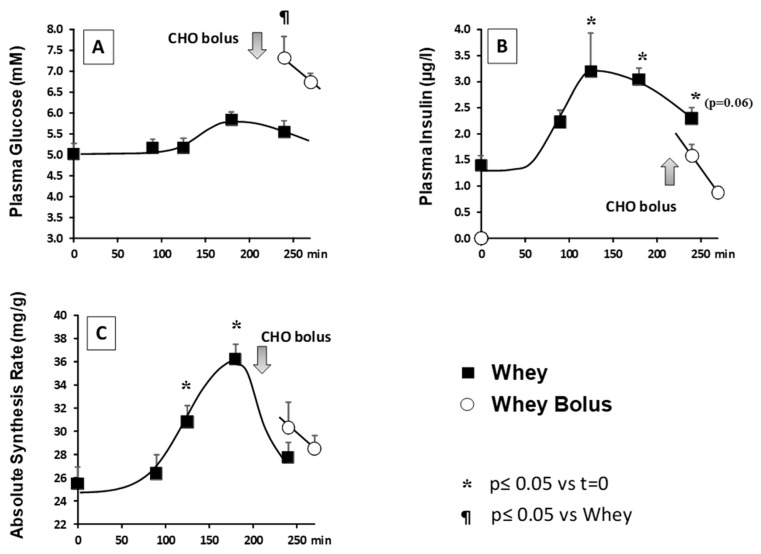
Plasma glucose (**A**), insulin (**B**) and muscle protein synthesis (**C**) in aged rats fed with whey or with whey followed by an energy bolus (CHO) 210 min after food intake. Data are presented as means ± SEM. (*n* = 10).

Plasma glucose increased significantly after the ingestion of the whey protein meal and glycemia peaked 180 min after food intake (Figure 2A). Insulin was also significantly increased after food intake to reach maximal values at 125 and 180 min (Figure 2B). When the energy bolus was given at 210 min, plasma glucose remained elevated and was significantly increased when compared to the WHEY CONTROL Group (Figure 2A). At 240 and 270 min, plasma glucose was 31 and 21% significantly higher, respectively, than at 240 min without the sugar bolus (Figure 2B). By contrast, insulinemia was not significantly increased after the intake of the energy bolus and even tended to be lower (−31%) than the value recorded at 240 min in the WHEY CONTROL (Figure 2B). 

### 3.3. Muscle Protein Synthesis

Muscle protein synthesis was significantly stimulated after the ingestion of the whey protein meal and maximum stimulation was recorded at 180 min (Figure 2C). After 180 min, muscle protein synthesis decreased to become similar to the post-absorptive value at 240 min. When the energy bolus was given at 210 min, it did not prevent a decrease in muscle protein synthesis in the Whey Bolus group and its value was not significantly different from the values recorded in the Whey group (Figure 2C).

## 4. Discussion

We previously showed that casein was unable to initiate the stimulation of muscle nitrogen balance or muscle protein synthesis when given at the amount fulfilling healthy adult requirements in the two anabolic resistant states presented, i.e., glucocorticoids treatment and aging [9,10]. The reason for this anabolic resistance was explained by the lower sensitivity of the main signaling pathway (i.e., mTOR signaling pathway), which leads to the stimulation of muscle protein synthesis in response to food intake and particularly to dietary leucine intake and availability [30,31,32,33]. These observations, which are now well accepted, have led to recommending in such situations the preferential intake of rapidly digested and leucine-rich proteins such as whey proteins. Indeed, when the availability of essential amino acids and/or leucine is increased, the defect in the stimulation of muscle protein synthesis is attenuated or reversed during both glucocorticoid treatment [33] and aging [34]. Long-term supplementation of free leucine or leucine-rich proteins was therefore tested in both elderly humans and aged rodents. Verhoeven et al. [16] tested a 3-month leucine supplementation (7.5 g/day) and showed that it did not augment skeletal muscle mass or strength in healthy elderly men. Animal studies also showed no beneficial effect of leucine or whey protein supplementation on muscle mass in aged rodents unless it was given in high amounts within a high protein diet [15]. In a recent review, Woo et al. [35] concluded that to date, evidence suggests that nutritional intervention including high-quality protein or leucine does have benefits in the elderly, but mainly if combined with exercise. Similar conclusions could be drawn from glucocorticoid-induced anabolic resistance in which leucine supplementation remained without effect against the deleterious effect of dexamethasone on muscle fiber atrophy and strength loss [18]. We previously showed that whey proteins could initiate an anabolic response in the skeletal muscle in glucocorticoid-treated minipigs, but that the response remained transient and was only visible during the early post-prandial period (i.e., first 2 h), in contrast to the 7 h-stimulated muscle protein accretion obtained in healthy conditions [10]. In other catabolic states, with muscle wasting and anabolic resistance, such as bed rest and immobilization, leucine supplementation and whey proteins supplementation have also been shown as not being optimal for preserving muscle mass and strength [19,36,37,38,39]. 

Recently, it has been shown that muscle protein synthesis stimulation after feeding is of finite duration even if amino acid availability is still high and mTOR signaling pathways remain activated [21,22]. It has been postulated that muscles can sense they are “full,” and this phenomenon has been named the “muscle full” effect [40,41]. Few studies have been carried out to elucidate the mechanisms involved in this “muscle full” effect and whether it can be prevented in order to prolong the anabolic response after food intake. They found that it was the decrease in muscle energy status associated with the increased activity of the intracellular energy sensor AMPK, which leads to blocking the stimulation of muscle protein synthesis [24,42]. However, when post meal supplements of carbohydrates (increased available energy) were given, the activation of AMPK was prevented, and the post-prandial muscle anabolic response was significantly extended in young growing rodents [24,42]. This could constitute a new strategy for slowing down muscle loss during the catabolic state.

Therefore, for the first time, we tested this nutritional strategy in catabolic models: glucocorticoid-treated young pigs and aging rats (a milder anabolic resistant state). In glucocorticoid treated pigs, the carbohydrate bolus further increased plasma glucose and insulin levels but remained without effect on the duration of muscle protein synthesis stimulation. Similarly, in aging rats, no modification in the duration of this stimulation was recorded. We hypothesize that glucocorticoids, which generate insulin resistance at the skeletal muscle level, had altered glucose uptake and metabolism and then rendered the energy bolus inefficient for maintaining or increasing ATP during the remaining postprandial period. In the aged rodents, it is noteworthy that our energy bolus dramatically increased plasma glucose, as expected, but did not increase plasma insulin. Since this increase of insulin was recorded in younger animals in which the energy bolus prolonged muscle protein synthesis, it is possible that during aging the lack of insulin elevation after the carbohydrate bolus did not allow simultaneous glucose uptake and metabolism in skeletal muscles and was unable to correct the energy deficit and AMPK activation. This is consistent because it has been shown that elderly subjects have a lower pulse amplitude and less responsive insulin secretion regarding oscillations in glucose (see [43] for a review). Furthermore, as mentioned for the glucocorticoid treatment, aging is also associated with glucose intolerance/insulin resistance, which may have further prevented glucose uptake during the energy bolus intake.

In addition, specific defects in AMPK pathways are possible. Indeed, it was shown that dexamethasone treatment caused intracellular ATP deprivation and robust AMPK activation [44]. This could explain the lack of effect of the energy bolus, which is assumed to increase ATP availability and reduce AMPK activation. Similarly, aging can disrupt AMPK signaling. For instance, mitochondrial biogenesis is reduced during aging in muscle, in particular in response to alteration in initial signaling through AMPK [45].

Other methodological differences exist between our study and the studies examining the effects of leucine or carbohydrate supplements for regulating protein synthesis duration [23,24]. The young mature rats were trained to consume three meals per day and were slightly food restricted (−20%). Since AMPK is a major sensor of energy availability in tissues, this food restriction could stimulate AMPK activity. Our older rats were not food-restricted and had much higher stores of energy than the young adult rats, which could also explain the lack of effect of our energy bolus on protein synthesis.

### Limiting Points of the Study

In the case of the glucocorticoid-treated minipigs, we may also hypothesize that, contrary to rodents, the CHO bolus was unable to prolong the duration of the anabolic response to food intake in healthy control animals fed a whey protein meal. We did not perform trials with this group in the present study, so we cannot exclude that the lack of effect of the CHO bolus was not related to the glucocorticoid treatment *per se* but was possibly also related to species-specific metabolic response to this nutritional strategy. Another possibility is that since glucocorticoid treatment reduced the overall response of muscle protein synthesis to feeding, and in particular led to an earlier peak of protein synthesis, our energy bolus could have been given too late to achieve the stimulation of muscle protein synthesis in this group. Finally, for technical reasons, we were unable to measure AMP/ATP and/or P-AMPK/AMPK ratios, which would have been a beneficial addition to our study. However, we had already shown that in both the models tested, resistance to the anabolic effect of food intake was indeed correlated with a defect in the activation of the Akt/mTOR signaling pathway, and thus the energy bolus was a pertinent strategy for trying to prevent this resistance and prolong its duration [9,10]. Further studies are necessary to confirm that the “energy bolus strategy” is not possible in catabolic situations.

## 5. Conclusions

The use of a simple energy bolus to prolong the anabolic response to a meal would have been a very interesting tool for preventing muscle loss during catabolic states. The carbohydrate bolus was indeed efficient in young rats fed whey protein meals to sustain muscle protein synthesis [24]. However, our attempt to prolong the already weak anabolic effect of whey in our catabolic model and to increase the anabolic muscle response of whey during aging using a desynchronized carbohydrate bolus during the postprandial period did not succeed. We emphasize that nutritional strategies aimed at preventing the adverse effect of catabolic states on skeletal muscle requires additional studies and would probably need a combination of several levers, including physical activity.
